# Blockade of HMGB1 signaling pathway by ethyl pyruvate inhibits tumor growth in diffuse large B-cell lymphoma

**DOI:** 10.1038/s41419-019-1563-8

**Published:** 2019-04-15

**Authors:** Tian Zhang, Xu-Wen Guan, John G. Gribben, Feng-Ting Liu, Li Jia

**Affiliations:** 1 Department of Radiotherapy, National Clinical Research Center for Cancer, Key Laboratory of Cancer Prevention and Therapy, Tianjin Medical University Cancer Institute and Hospital, Tianjin, China; 2 Tianjin Medical University, Tianjin, China; 3 Centre for Haemato-Oncology, Barts Cancer Institute, Queen Mary University of London, Charterhouse Square, London, UK; 4 Department of Hematology and Oncology, Tianjin Union Medical Center, Tianjin, China

## Abstract

High mobility group box 1 (HMGB1) protein in the tumor microenvironment actively contributes to tumor progression but its role in diffuse large B-cell lymphoma (DLBCL) is unknown. The aim of this study was to determine the mechanism by which HMGB1 promotes tumor growth in DLBCL and whether blockade of HMGB1 signaling pathway could inhibit tumorigenesis. We report that HMGB1 promotes proliferation of DLBCL cells by activation of AKT, extracellular signal-regulated kinases 1/2 (ERK1/2), signal transducer and activator of transcription 3 (STAT3) and SRC Proto-Oncogene, Non-Receptor Tyrosine Kinase (Src). Ethyl pyruvate (EP), an anti-inflammatory agent, inhibits HMGB1 active release from DLBCL cells and significantly inhibited proliferation of DLBCL cells in vitro. Treatment with EP significantly prevented and inhibited tumor growth in vivo and prolonged DLBCL-bearing mice survival. EP significantly downregulated HMGB1 expression and phosphorylation of Src and ERK1/2 in mice lymphoma tissue. EP induced accumulation of the cell cycle inhibitor p27 but downregulated expression of cyclin-dependent kinase 2 (CDK2). Increased nuclear translocation of p27 interacted with CDK2 and cyclin A, which led to blockade of cell cycle progression at the G1 to S phase transition. In conclusion, we demonstrated for the first time that blockade of HMGB1-mediated signaling pathway by EP effectively inhibited DLBCL tumorigenesis and disease progression.

## Introduction

Diffuse large B-cell lymphoma (DLBCL) is one of the most common forms of aggressive non-Hodgkin lymphomas (NHLs). Treatment with chemotherapy achieved high response rates and led to significant improvements on overall survival rates in patients with NHLs. However, there are still about 30% DLBCL patients who currently remain incurable with conventional chemotherapy^1^. It is characterized by highly biological heterogeneity which is caused not only tumor cells themselves but also dependent on the tumor microenvironment^2–4^. The more aggressive type of DLBCL, active B cell-like (ABC), has constitutively activated NF-κB and STAT3 tumor survival signaling pathways compared with the germinal center B-cell (GCB) subtype^4–7^. Considering the limited treatment options currently available for ABC-DLBCL and the poor prognosis for patients with recurrent disease, new therapeutics and diagnostics are urgently required^6^.

Cytokines including inflammatory factors in the microenvironment support tumor cell proliferation and survival^8,9^. Many inflammatory factors promote tumor growth through Toll-like receptor (TLR)-mediated signaling pathways, which lead to activation of PI3/AKT, ERK, Src, NF-κB, and STAT3^10–13^. Stressed, injured or dying cells release damage-associated molecular patterns (DAMPs), which initiate noninfectious inflammatory responses^14–17^. HMGB1 (high mobility group B1) protein, one of the DAMPs, is released from damaged, inflamed, and tumor cells which in turn promotes tumor cell survival^17–21^. In most human cells, HMGB1 is located in the nucleus, where it acts as a DNA chaperone to help maintain nuclear homeostasis. HMGB1 has many biological functions inside as well as outside of the cell, especially promoting inflammation and tumorigenesis^22–24^. HMGB1 can be actively secreted by innate immune cells in response to pathogenic products or passively released by injured and necrotic cells^25,26^. However, the role of extracellular HMGB1 in DLBCL is still unknown.

Ethyl pyruvate (EP) is a nontoxic food additive and has a function to counteract with HMGB1. It has been shown highly effective in the in vivo treatment of severe inflammation and several types of cancers in mice models^27–32^. EP treatment significantly reduces circulating levels of HMGB1 in mice with established sepsis^28^ or colitis^31^, suggesting that EP inhibits HMGB1 release from the cell. However, the precise mechanism by which EP inhibits tumor growth is elusive.

We previously reported that higher levels of extracellular HMGB1 is associated with poor clinical outcome in patients with chronic lymphocytic leukemia (CLL)^20^. In this study, we aimed to determine the signaling pathway of extracellular HMGB1 and its roles in tumor proliferation in both ABC-DLBCL and GCB-DLBCL. We hypothesized that targeting HMGB1 using EP treatment could inhibit DLBCL tumor growth. Here, we report for the first time that treatment with EP significantly inhibited DLBCL tumor growth in vitro and in vivo by blockade of HMGB1-mediated Src/ERK signaling pathway and cell cycle G1 to S phase transition.

## Results

### HMGB1 stimulates proliferation of GBC-type DLBCL cells

**Fig. 1:**
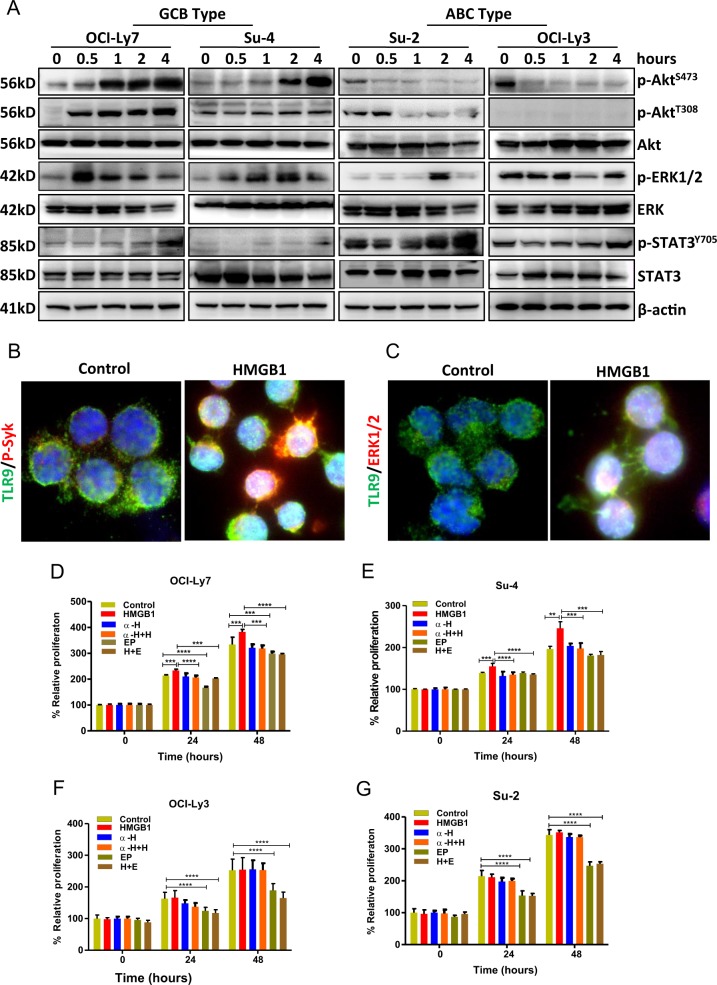
Effect of HMGB1 or EP on cell proliferation. **a** HMGB1-mediated activation of survival pathway in GCB-DLBCL cells. GCB and ABC-DLBCL cell lines were stimulated with 200 ng/ml HMGB1 for indicated periods and the activation of survival signaling molecules AKT, ERK, and STAT3 was determined by western blotting. Detailed information for primary and secondary antibodies was listed in Suppl Table 1. **b** Fluorescent microscopy of HMGB1-mediated TLR9 activation. The GCB-DLBCL cell line Su-DHL4 was stimulated with 200 ng/ml human recombinant HMGB1 for 24 h. Costained primary antibodies used were mouse anti-TLR9 (green)/rabbit anti-p-Syk (red) (top panel) and mouse anti-TLR9 (green)/rabbit anti-p-ERK(1/2) (red) (bottom panel) antibodies. Secondary antibodies for costaining were Alexa Fluor-488 anti-mouse IgG and Alexa Fluor-546 anti-rabbit IgG. **c**–**g** GCB-DLBCL cell lines Ly-7 and Su-4, and ABC-DLBCL cell lines Ly-3 and Su-2 were treated with 200 ng/ml HMGB1, 10 µg/ml anti-HMGB1 antibody (α-H), both HMGB1 and anti-HMGB1 antibody (α-H+H), 2-mM EP, or both HMGB1 and EP (H+E) for 24 and 48 h. Cell proliferation was determined by the MTT assay. Data presented were from three independent experiments. Proliferation rates were expressed as mean ± SD and the significant differences were analyzed by the student *t*-test

Signaling through AKT, ERK, and STAT3 pathways controls cell proliferation and these molecules are constitutively phosphorylated in ABC-DLBCL (OCI-Ly3 and Su-2) but not in GCB-DLBCL (Su-4 and OCI-Ly7) cell lines (Suppl Fig. 1A). We determined whether extracellular HMGB1 could stimulate proliferation of DLBCL cells. DLBCL cell lines were treated with 200 ng/ml human recombinant HMGB1 protein. After stimulation with HMGB1 for 0.5–4 h, increased phosphorylation of AKT (both p-AKT^S473^ and p-AKT^T308^) and ERK(1/2) was observed mainly in GCB-DLBCL cell lines, although increased phosphorylation of p-STAT3^Y705^ was seen in both subtypes of DLBCL cells (Fig. 1a). HMGB1 promotes tumor cell proliferation via multiple TLR receptors, mainly TLR4, TLR9, and advanced glycosylation end-product specific receptor (RAGE)^33,34^. TLR4 is mainly expressed in monocytes but not in B-cells^35^, therefore the role of HMGB1 on TLR4 in DLBCL cells was excluded in this study. Stimulation of GCB-DLBCL cells with human HMGB1 led to TLR9 redistribution and colocalization with phosphorylated Syk and ERK(1/2), as detected by fluorescent microscopy (Fig. 1b), suggesting that HMGB1 activates the TLR9 pathway in DLBCL cells. The role of HMGB1 on RAGE redistribution in DLBCL cells was not detected (data not shown).

EP is an inhibitor for HMGB1 release and also downregulates HMGB1 expression which are both upstream events of HMGB1-mediated signaling pathways^28,36^. To prove the effect of HMGB1, 10 µg/ml neutralizing anti-HMGB1 antibody or 2-mM EP was used alone or combined with HMGB1 in this setting. DLBCL cell proliferation was determined using both MTT test and cell number assays after treatment for 24 and 48 h. Incubation with HMGB1 protein significantly increased cell viability and cell numbers in GBC but not in ABC type cell lines. The anti-HMGB1 antibody completely prevented HMGB1-induced increase in cell proliferation in GCB cell lines. However, treatment with 2 mM of EP significantly decreased cell viability and cell number in both GBC and ABC type cell lines (Fig. 1d–g and Suppl Fig. 1B–E), indicating that HMGB1-mediated cell survival signaling was constitutively activated in the ABC type DLBCL cells. These results demonstrate that extracellular HMGB1 promotes proliferation of GBC but not ABC type DLBCL cells but inhibition of HMGB1 by EP reduced proliferation in both types of DLBCL cells.

### Treatment with EP prevents and inhibits lymphoma tumor growth and prolongs survival of lymphoma-bearing mice

**Fig. 2:**
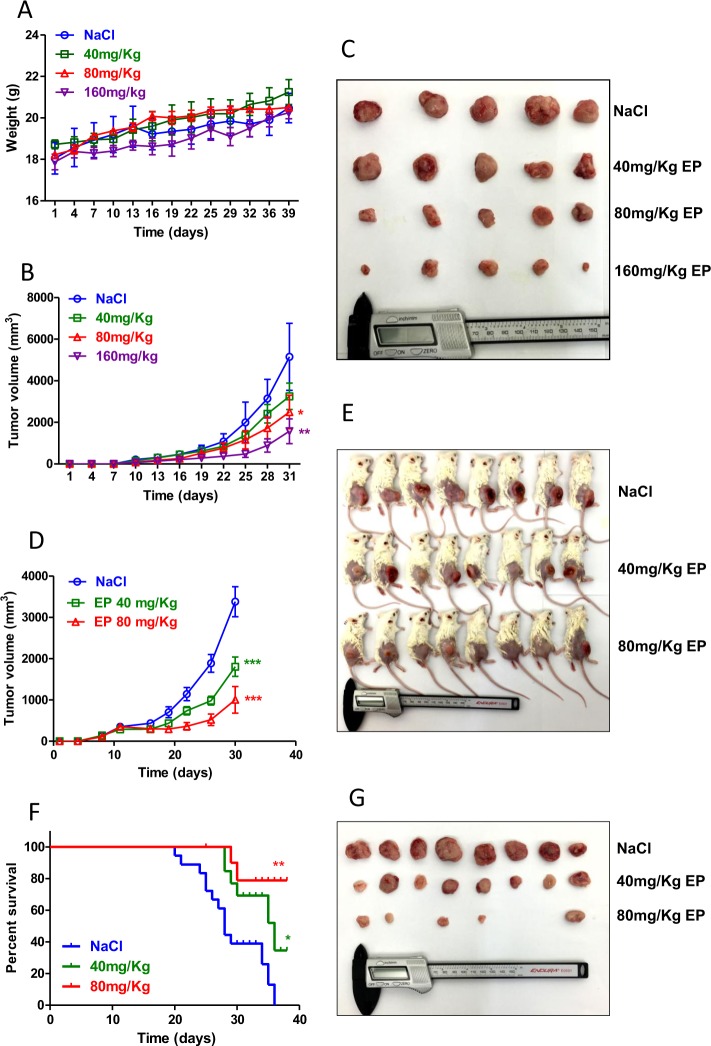
Inhibitory effect of EP on tumor growth in lymphoma-bearing mice. **a** Toxicity assay of EP in wild-type BALB/C mice. **b**, **c** After intraperitoneal injection of NaCl, 40 mg/kg EP, 80 mg/kg EP or 160 mg/kg EP twice/per day for 39 days, A20 cells were injected into mice subsequently and tumor volume was measured in every 3 days. **e** Killed mice after treatment; **d**, **e**, **g** A20 cells were inoculated into female BALB/C mice. When tumors were palpable, mice were randomly divided into three groups and undergone intraperitoneal injection of NaCl, 40 mg/kg EP, or 80 mg/kg EP twice/per day for 30 days. Effect of EP on tumor volumes was analyzed by ANOVA. **f** The Kaplan–Meier survival curves for each group of lymphoma-bearing mice were analyzed by the log rank test

To determine whether HMGB1 antagonist EP could prevent DLBCL proliferation in vivo, we first conducted the EP toxicity assay in wild-type mice. Young BALB/C mice with similar weight were randomly and equally divided into four groups (four mice/group). Mice were intraperitoneal injected twice/per day with NaCl, 40, 80 or 160 mg/kg of EP, respectively for 39 days and weighed every 3 days. Treatment with EP did not show any effect on mice weight compared with the control group (Fig. 2a). The cytotoxicity of EP on mice B-cell lymphoma A20 cells was determined by the MTT test. The IC50 of EP after 48 h treatment was 3.67 mM in vitro (Suppl Fig. 2), equivalent to 360 mg/kg EP in vivo. To test whether EP has preventive role in tumorigenesis, A20 cells were injected into mice subsequently after withdrawing EP treatment. Tumor volume in mouse was measured every 3 days. The results showed that mice treated with 80 and 160 mg/kg EP significantly reduced tumor growth compared with the control group (Fig. 2b, c). We then investigated whether EP could directly inhibit DLBCL tumor growth in vivo. Mice A20 cells were inoculated into female BALB/C mice. When tumors were palpable, mice were randomly and equally divided into three groups (eight mice/group) and undergone intraperitoneal injection of NaCl, 40, or 80 mg/kg EP twice/per day for 30 days. Tumor volumes in 40 and 80 mg/kg EP-treated mice were significantly reduced (Fig. 2d, e, g). Strikingly, tumors in part of 80 mg/kg EP-treated mice were completely vanished (Fig. 2e, g). Most important, EP-treated mice showed significantly prolonged survival rates (Fig. 2f). FDA approved EP in cold beverages for human is ∼150 mg/kg, indicating that the doses of EP used in mice are achievable in human. These results indicate that treatment with EP prevents and inhibits lymphoma tumor growth and therefore prolongs survival of lymphoma-bearing mice.

### EP inhibits HMGB1 release and decreases HMGB1 protein expression

**Fig. 3:**
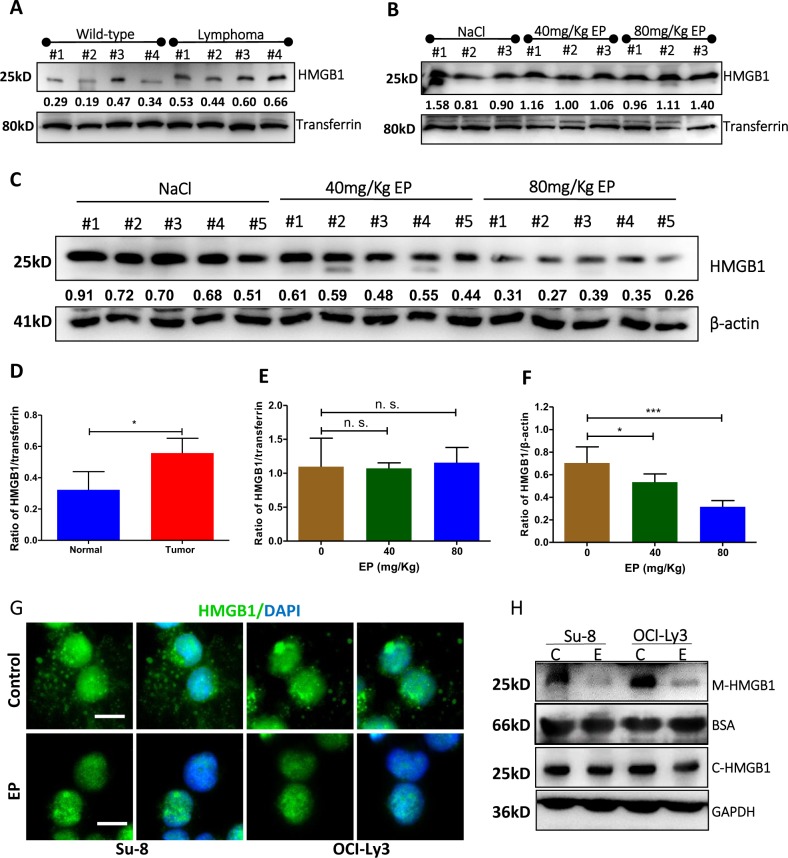
Inhibitory effect of EP on HMGB1 release and expression. **a,** **d** Extracellular HMGB1 in the plasma of wild-type mice and lymphoma-bearing mice. **b**, **e** Extracellular HMGB1 in the plasma of lymphoma-bearing mice treated with 40 or 80 mg/kg EP. Mice injected with NaCl were used as control. Transferrin was used as loading control for plasma. **c**, **f** HMGB1 expression in lymphoma tissue. β-actin was used as loading control for cells. **g**, **h** Effect of EP on HMGB1 release in vitro determined by immunofluorescent microscopy (**g**) and western blotting (**h**). After cultured for 6 (Su-8) or 3 (Ly-3) h, DLBCL cells and medium were separately collected. After fix/permeabilization, cells were stained with rabbit anti-HMGB1 antibody, showing green color. DAPI staining (blue) indicates the nuclear localization. The green dots in the background indicate a typical phenomenon of HMGB1 release from the cells. Scale bar: 10 µm. Extracellular HMGB1 in the medium (M-HMGB1) and HMGB1 expression in cell lysate (C-HMGB1) were determined by western blotting. BSA and GAPDH were used as loading control. Numbers below each pair of bands are radios of interest proteins to the loading controls analyzed by densitometry. The significant differences (d–f) were analyzed by the student *t*-test

It was previously reported that EP prevents mice lethal sepsis by inhibiting HMGB1 release from inflammatory cells^28^. We aimed to determine whether this is applied to lymphoma-bearing mice. Blood plasma and tumor tissue in lymphoma-bearing mice were collected at the endpoint to determine whether EP could reduce levels of HMGB1 in the plasma and/or alter specific protein expression. The levels of extracellular soluble HMGB1 in mice plasma were determined by western blotting. The levels of HMGB1 in the plasma of lymphoma-bearing mice were significantly higher compared with wild-type mice (Fig. 3a, d). Surprisingly, plasma HMGB1 levels in EP-treated mice were similar to the control group (Fig. 3b, e). This may be due to HMGB1 passive release by dead tumor cells after treatment with EP. We then tested HMGB1 protein expression in lymphoma tumor tissue by western blotting. In both 40 and 80 mg/kg EP-treated mice, HMGB1 expression in lymphoma tissue was significantly decreased compared with untreated mice (Fig. 3c, f). This result suggests that treatment with EP inhibits HMGB1 expression.

To confirm whether EP inhibits HMGB1 release, we did in vitro experiment using EP to treat GCB-DLBCL cell line Su-8 and ABC-DLBCL cell line OCI-Ly3. Release of HMGB1 from cells was determined by fluorescent microscopy and western blotting. We found that ABC type OCI-Ly3 cells started to release HMGB1 after 3 h in culture medium and GCB-type Su-8 cells release HMGB1 after 6 h period, as shown the green dots (HMGB1) observed by fluorescent microscopy (Fig. 3g) and HMGB1 positive band detected by western blotting (Fig. 3h). When cells were treated with 2-mM EP, HMGB1 release was completely inhibited (Fig. 3g, h). However, EP could not inhibit HMGB1 release from cells after 24 h treatment, suggesting that passive HMGB1 release is involved in this process. Taken together, these findings demonstrate that EP inhibits HMGB1 active release and reduces HMGB1 protein expression in lymphoma-bearing mice.

### Low dose EP induces cell cycle arrest at G1 phase

**Fig. 4:**
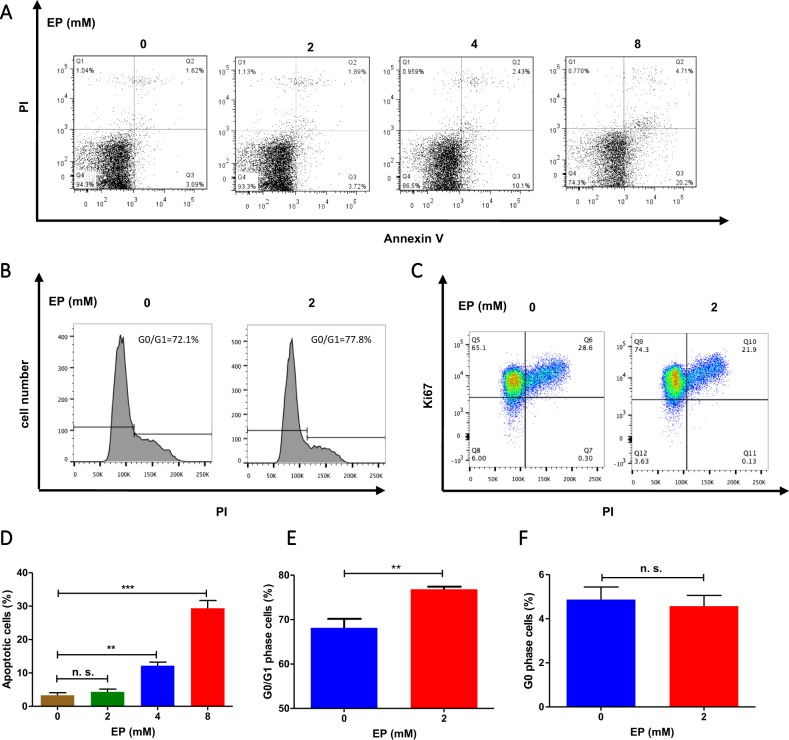
Flow cytometry analysis of EP on apoptosis and cell cycle progression. ABC-DLBCL Su-2 cells were cultured with or without indicated concentrations of EP for 24 h. **a**, **c** Apoptotic cell death was measured by flow cytometry after staining with annexin V-FITC and PI. **b**, **d** Cell cycle distribution analysis by quantitation of DNA content. Cells were stained with PI and DNA content was analyzed by flow cytometry. **e**, **f** Cell cycle analysis with Ki67 and PI. Permeabilized cells were stained with Ki67-FITC and PI and DNA content was analyzed by flow cytometry. The percentages of cells within each cell cycle phase are depicted by density plots. **c**, **d**, **f** Data shown (mean ± SD) are from three independent experiments. The significant difference between EP-treated and control was analyzed by the student *t*-test

The doses of EP given to mice were 40 and 80 mg/kg, which equal to 0.8 and 1.6-mM EP in vitro. To further examine the mechanism how EP inhibits lymphoma growth, both ABC (Su-2) and GCB (Su-8) cells were treated with 2, 4, and 8-mM EP for 24 h. Apoptotic cell death was determined by flow cytometry using the annexin V-FITC/PI kit. Low dose of EP (2 mM) which close to 80 mg/kg in vivo did not significantly increase percentages of apoptotic cells tested in either Su-2 or Su-8 cells (Fig. 4a, c; Suppl Fig. 3A, C). DNA content analysis revealed that treatment with 2-mM EP significantly induced cell cycle arrest in G0/G1 phase (Fig. 4b, d; Suppl Fig. 3B, D). Ki67 is a cellular maker for proliferation, which is expressed throughout the cell cycle, except for the G0 phase^9^. We aimed to differentiate whether EP induces cell cycle arrest in G0 or/and G1. Flow cytometry of Ki67 staining profiles showed that EP-treated cells did not increased percentage cells in the G0 phase compared with untreated group, indicating that cell cycle arrested in the G1 phase (Fig. 4e, f; Suppl Fig. 3E). Cell cycle arrest in the G1 phase induced by 2-mM EP is consistent in both Su-2 and Su-8 cell lines. However, higher concentration of EP (8 mM) induced cell cycle arrest in G2/M (Suppl Fig. 3B, F). These data demonstrated that EP might inhibit DLBCL cell proliferation by inducing cell cycle arrest in the G1 phase.

### Inhibition of the Src/ERK pathway by EP induces upregulation of G1 phase inhibitor p27

**Fig. 5:**
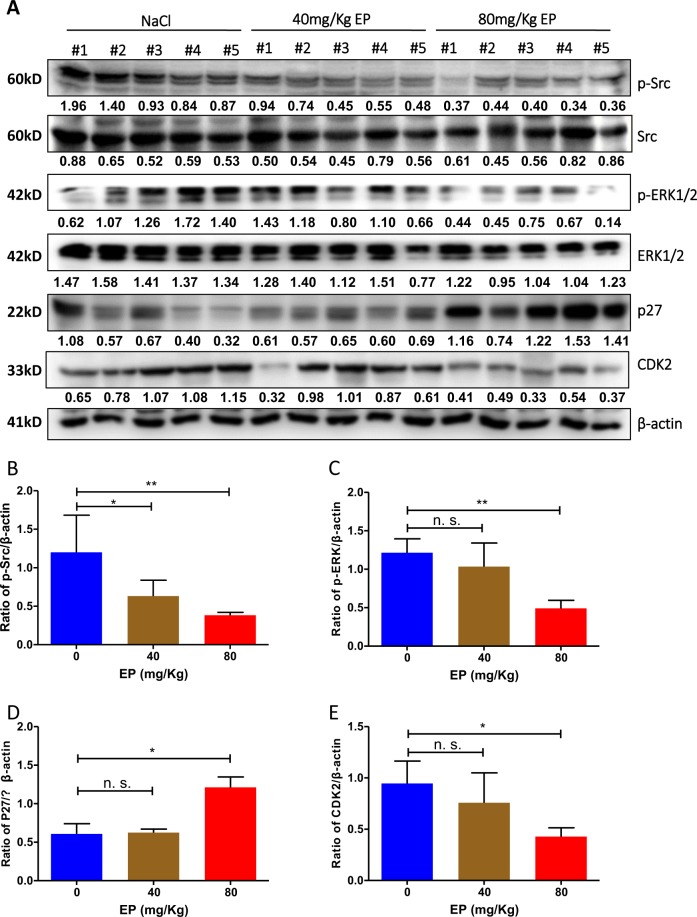
Effect of EP on phosphorylation of Src/ERK and levels of p27. Tumor cells were isolated from solid tumor by separating cells from connective tissue. Fifteen lymphoma tissues from NaCl, 40 mg/kg and 80 mg/kg EP injected, five in each group were lysed for western blotting. **a** Western blotting analysis of the effect of EP on p-Src, p-ERK, CDK2, and p27. Condition of each antibody is listed in Suppl Table 1. **b**, **c** Statistical analysis of EP-induced inhibition of p-SRC and p-ERK. **d**, **e** Statistical analysis of EP-induced inhibition of CDK2 and accumulation of p27. Statistical analyses were as same as mentioned in Fig. 3

Src is a non-receptor tyrosine kinase that is deregulated in many types of cancer. Src plays crucial role in many aspects of tumor development, including proliferation, survival, adhesion, migration, invasion, and metastasis^37^. To investigate the mechanism of the EP-induced G1 phase arrest in DLBCL cells, western blot analyses were conducted on various cell cycle regulators both in vitro and in vivo. Phosphorylation of Src and ERK plays important roles in tumor cell proliferation and ERK is a downstream signaling molecule of Src^38,39^. Src kinase destabilizes p27, an inhibitor of G1 cyclin-CDK protein kinase^40–42^. In mice tumor tissue, phosphorylation of Src and ERK1/2 and expression of CDK2 were significantly decreased in EP-treated groups (Fig. 5a–c, e). On the contrary, expression of p27 was significantly increased by the treatment with EP (Fig. 5a, d). Taken together, we demonstrate that treatment with EP inhibits phosphorylation of Src and ERK1/2 but increases expression of p27. EP-induced G1 phase arrest is p27-dependent.

### Treatment with EP induces protein interaction between p27 and cyclin A/CDK2

**Fig. 6:**
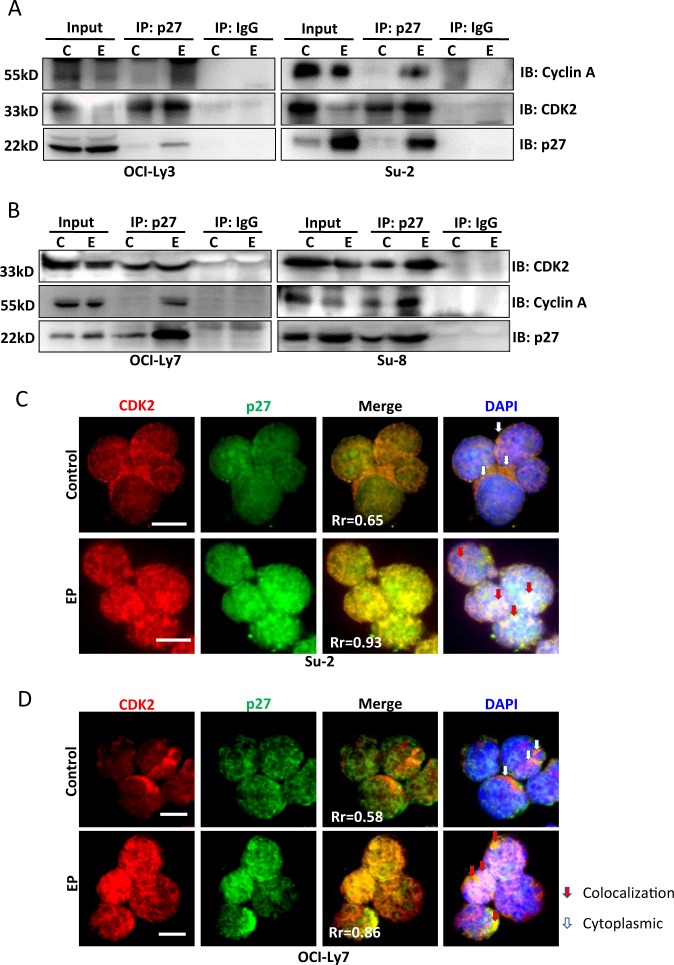
EP-induced interaction between P27 with cyclin A and CDK2. ABC-DLBCL cells Ly-3 and Su-2 (**a**) and GCB-DLBCL cells Su-8 and Ly-7 (**b**) were treated with 2-mM EP for 24 h. **a**, **b** Protein complex was immuno-precipitated with a rabbit anti-p27 antibody and blots were probed with either a mouse anti-CDK2, a mouse anti-cyclin A, or a rabbit anti-p27 antibodies, respectively. **c** Immunofluorescent determination of colocalization of p27 and CDK2 in Su-2 and Ly-7 cells. After treatment, cells on slides were costained with mouse anti-CDK2 (red)/rabbit anti-p27 (green) antibodies. Secondary antibodies for costaining were FITC-conjugated goat anti-rabbit IgG and Rhodamine-conjugated goat anti-mouse IgG. White arrows indicate cytoplasm and red arrows indicate colocalization of p27 and CDK2. Colocalization coefficient (Rr) was analyzed by the intensive correlation analysis program using WCIF ImageJ software. Scale bar: 10 µm

Using immunoprecipitation, we found that treatment with EP remarkably increased protein interaction between p27 with cyclin A and CDK2 in both ABC (Fig. 6a) and GCB-type cells (Fig. 6b). In untreated cells, most p27 and CDK2 were localized in the cytoplasm with less colocalization (Rr = 0.58–0.65), although weakly expressed in the nucleus. Treatment with EP increased nuclear translocation and colocalization of p27 and CDK2 (Rr = 0.85–0.93) (Fig. 6c, d; Suppl Fig. 4A, B). These results demonstrate that EP induces cell cycle arrest by increasing binding between p27 and cyclin A/CDK2.

### HMGB1-mediated cell cycle progression is Src dependent

**Fig. 7:**
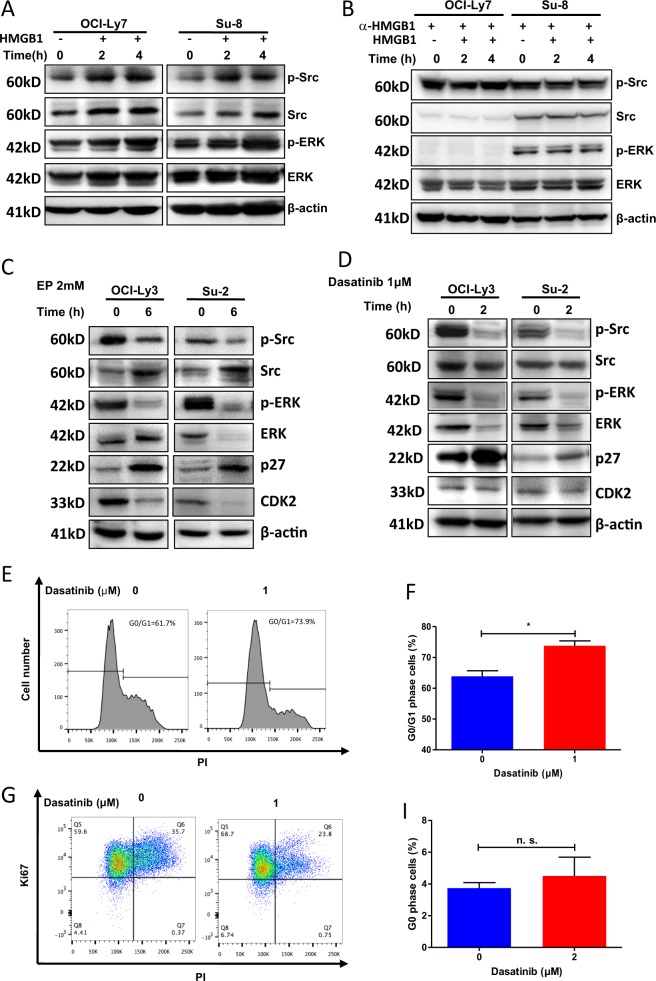
The role of SRC phosphorylation in HMGB1-mediated cell cycle progression. **a**, **b** Effect of HMGB1 on phosphorylation of Src and ERK. GCB-DLBCL Ly-7 and Su-8 cell lines were incubated with 200 ng/ml HMGB1 alone (**a**) or with or without 10 µg/ml neutralizing anti-HMGB1 antibody (**b**) for indicated hours. Phosphorylation of Src and ERK was evaluated by western blotting. **c** Effects of EP on phosphorylated Src and ERK and levels of CDK2 and p27 in ABC-DLBCL Ly-3 and Su-2 cells. Cells were treated with 2-mM EP for 6 h. **d** Effect of Src inhibitor Dasatinib on phosphorylated Src and ERK, and levels of CDK2 and p27 in ABC-DLBCL cells. Cells were treated with 1 µM Dasatinib for 2 h. **e**, **f** Dasatinib-induced G0/G1 arrest. Su-2 cells were treated with Dasatinib for 24 h. Cell cycle was analyzed by DNA content analysis. **g**, **i** Effect of Dasatinib on G1 arrest. Cells were stained with Ki67 and PI. G0 phase was defined as the Ki67 negative population

We questioned whether EP-induced cell cycle arrest is mediated by inhibition of HMGB1. Incubation with HMGB1 induced phosphorylation of Src and ERK1/2 in GCB-type cells (Fig. 7a) and this event is prevented by using a neutralizing HMGB1 antibody (Fig. 7b). On the contrary, EP inhibited Src and ERK1/2 phosphorylation, increased the expression of p27 and inhibited the expression of CDK2 in ABC type cells (Fig. 5c, Suppl Fig. 5A). To confirm the link between Src with cell cycle, we treated ABC Su-2 and Ly-3 cells with Src inhibitor Dasatinib. Dasatinib-reduced phosphorylation of Src and ERK1/2 and increased p27 expression were identical to the results treated with EP (Fig. 7d, Suppl Fig. 5B). Furthermore, treatment with Dasatinib also induced G0/G1 phase arrest (Fig. 7e, f, Suppl Fig. 5C, D). Ki67 staining demonstrated that treatment with Dasatinib led to a significant increase in G1 phase cells but not G0 phase (Fig. 7g, i, Suppl Fig. 5E, F). These data demonstrated that HMGB1-mediated cell cycle progression may be via the activation of Src/ERK1/2.

## Discussion

Here we demonstrate for the first time that extracellular HMGB1 stimulates proliferation of GCB-DLBCL cells by activating Src/ERK pathway. In vivo treatment with EP significantly inhibited DLBCL tumor growth and prolonged survival of lymphoma-bearing mice through inhibition of HMGB1 expression and phosphorylation of Src and ERK1/2. Therefore, downregulated Src/ERK pathway caused an accumulation of p27 and cell cycle arrest in G1 to S phase transition.

It was recently reported that constitutively activated STAT3 and its kinase JAK1 is caused by autocrine production of IL-6 and IL-10 in the ABC-DLBCL subtype. Activated STAT3 regulates multiple oncogenic signaling pathways, including NF-κB, a cell cycle checkpoint, PI3K/AKT/mTORC1, and STAT3 itself^43^. Our results showed that AKT, Src, ERK, and STAT3 are constitutively activated in the ABC-DLBCL cell lines. Src is signaling molecule upstream of ERK and STAT3 and associates with chronic active B-cell receptor signaling. Dasatinib, an inhibitor of Src, inhibits proliferation of ABC-DLBCL but not GCB-DLBCL cells^44^. It is not surprising that HMGB1 promotes GCB-DLBCL cells proliferation but not the ABC-DLBCL cells, which oncogenic signaling pathway is constitutively active.

Increased plasma or serum levels of HMGB1 have been found in several cancers and higher circulating HMGB1 is associated with worse clinical outcomes in patients with CLL, colon carcinoma, or hepatocellular carcinoma^20,45,46^. In this study, we found that levels of HMGB1 in the plasma of lymphoma-bearing mice were significantly higher compared with the wild-type mice. In vitro experiments confirmed that viable DLBCL cells started to release HMGB1 after 3–6 h, suggesting that the active release of HMGB1 from DLBCL B-cells to the microenvironment could occur in DLBCL. However, it is unknown whether actively released HMGB1 remains in the tumor microenvironment or enters the blood stream.

HMGB1 active secretion requires posttranslational modification, such as hyper-phosphorylation and hyper-acetylation within HMGB1 nuclear localization signal domains^47,48^. EP inhibits HMGB1 active secretion via deacetylation of HMGB1^49^. Although, treatment with EP inhibited HMGB1 release in vitro, levels of plasma HMGB1 in EP-treated lymphoma mice were not significantly decreased by comparison with non-EP-treated lymphoma mice. This suggests that treatment-mediated killing on tumor cells causes HMGB1 passive release from dead cells. We previously reported that treatment-mediated release of HMGB1 has function to stimulate immune function of dendritic and T-cells for eliminating tumor cells^50^. Nevertheless however, the precise mechanisms by which actively or passively released extracellular HMGB1 functions on tumor cells’ fate remain unclear.

The anticancer properties of EP have been demonstrated in several types of cancer in animal models^30–32,51^. The role of EP on DLBCL was not yet reported. The murine A20 cell line was implanted subcutaneously in BALB/C to develop lymphoma-bearing model. This model mimics aspects of DLBCL in humans^52^. However, it is unknown whether it is ABC or GCB type of DLBCL. We found that both treatment with EP significantly suppressed tumor growth in vivo and prolonged the survival of lymphoma-bearing mice. Interestingly, pretreatment with EP also inhibited tumor growth, suggesting that EP has preventive effect on lymphoma. As EP inhibits HMGB1 active release but not passive release, we examined the role of EP HMGB1 expression in tumors. Overexpression of HMGB1 in tumor is associated with poor prognosis in many cancers^53–55^. HMGB1 expression in DLBCL tumor tissue was significantly decreased in EP-treated lymphoma-bearing mice compared with untreated group. This indicated that EP counteracts with HMGB1 by inhibiting its active release and reducing its protein levels in the cell.

EP is not toxic to normal cells and the mechanism of EP to kill tumor cells is unclear. Our results demonstrated that low dose (less than 4 mM) EP did not directly kill DLBCL cells by inducing apoptosis but this treatment caused significant cell cycle arrest in the G1 phase. Different combinations of CDK and cyclin subunits operate at checkpoint controls during the cell cycle to integrate mitogenic and antiproliferative signals. Among the main players in mammalian cells are cyclins, CDKs, and CDK inhibitors (CKIs). Because a defect in CKI activity is one of the factors causing uncontrolled proliferation of tumor cells, one possible strategy to control cancer cell proliferation is to induce CKI expression, ultimately leading to cell cycle arrest and inhibition of tumor growth and p27 is one of the CKI in cancer^56^. We found that treatment with EP induced upregulation or accumulation of p27 but not other Cip/Kip family proteins (data not shown) and a reduction of cyclin A/CDK2 protein expression. This may explain EP-induced inhibition of tumor cell growth due to blocking G1–S phase transition in DLBCL cells. Using immunoprecipitation and immunofluorescence, we observed that treatment with EP significantly increased binding between p27 with cyclin A and CDK2. These data suggest that EP-induced cell cycle arrest may be a downstream effect of blockade HMGB1-mediated signaling pathway.

In tumor cells, downregulation of p27 requires activation of Src and ERK1/2^57,58^. To explore the link between inhibition of HMGB1 signaling pathway and cell cycle arrest, we examined whether EP-induced upregulation of p27 is associated with downregulation of Src and ERK1/2. HMGB1 induced phosphorylation of both Src and ERK1/2 in GCB-DLBCL cells. Src and ERK1/2 are both constitutively phosphorylated in ABC-DLBCL cells and mice DLBCL tissues. EP-mediated inactivation of Src and ERK1/2 accompanied by upregulation of p27 in tumor tissue and ABC-DLBCL cells. Inhibition Src by Dasatinib downregulate ERK1/2 in ABC-DLBCL cells, indicating that ERK1/2 is a downstream molecule of Src. Similar to the effect of EP, treatment with Dasatinib also induced upregulation of p27, downregulation of CDK2 and a cell cycle arrest in G1 phase.

In summary, we demonstrated for the first time that treatment with EP effectively prevents and inhibits DLBCL tumor growth. HMGB1-mediated tumor cell proliferation is dependent on phosphorylation of Src and ERK1/2 in lymphoma-bearing mice. Treatment with EP blocks HMGB1-mediated Src/ERK1/2 phosphorylation and leads to cell cycle arrest in G1 phase by upregulation of p27. EP is a low-cost anti-inflammation and anti-tumor agent. It has been commercially used as food additive, flavoring agent, due to nontoxicity. We propose that EP may have great potential in controlling DLBCL disease progression and reducing the treatment cost for cancer patients.

## Materials and methods

### Cell lines and cell culture

Human DLBCL cell lines Su-DHL-4 (Su-4), Su-DHL-8 (Su-8), and Su-DHL-10 (Su-10) were kindly provided by Professor Anthony Letai (Dana-Farber Cancer Institute). Human DLBCL cell lines OCI-Ly7 and TMD8 were kindly provided by Dr. Xiujuan Zhao (Tianjin Medical University). Human OCI-Ly3 and Su-DHL-2 (Su-2) were kindly provided by Professor Yizhuo Zhang (Tianjin Medical University Cancer Institute and Hospital). The mouse B-cell lymphoma cell line A20 (strain BLAB/cAnN)^59^ was purchased from the AACC Universal Sample Bank. The human GCB-type cell lines Su-4, Su-8, and Su-10^60^, mouse cell line A20, and the human ABC cell lines OCI-Ly3, Su-2, and TMD8^6,61,62^ were routinely cultured in RPMI-1640 medium. The human GCB Ly-7 cell line^61^ was cultured in IMDM medium. All culture media supplemented with 10% heat-inactivated fetal bovine serum (FBS), 2.0 mM L-glutamine, 100 U/ml penicillin, and 100 mg/ml streptomycin. All cells were cultured in a humidified incubator at 37 ℃ with 5% CO_2_.

### MTT assay

Cells were seeded in a 96-well plate at 4 × 10^4^ cells/well in 100 μl medium. After 24 or 48 h incubation, 10 μl of 5 mg/ml MTT (3-(4,5-dimethylthiazol-2-yl) -2,5-diphenyltetrazolium bromide, Sigma) was added to each well. After incubation at 37 ℃ for 4 h, tetrazolium was reduced by mitochondrial dehydrogenase to form purple colored formazan. The crystals were dissolved by adding 150 μl/well of isopropanol/0.04 N HCl and mixed well with a multichannel pipette. The optical density value of soluble formazan in each well was measured with a microplate reader at 570 nm (Thermo Scientific). Experiments were performed in triplicate.

### Western blotting

Cellular proteins were extracted by CellLytic M Cell Lysis Reagent (Sigma) supplemented with protease inhibitor and phosphatase inhibitor cocktails (Sigma). Protein concentration was determined by the Bradford method. The protein extract was mixed with NuPAGE LDS Sample Buffer (Invitrogen) and boiled for 5 min. Proteins (30 μg) were separated by 12% SDS-PAGE and transferred onto PVDF membrane (Sigma). PVDF membrane was blocked with the blocking buffer (Tris-buffered saline- 0.1% Tween-20 (TBST) containing 5% polyvinyl pyrrolidone (PVP), 5% FBS, and 0.1% sodium azide) for 1 h and then incubated with primary antibody overnight at 4 ℃^20^. After washing the membranes for three times, PVDF membrane was incubated with HRP-conjugated secondary antibodies (Cell Signaling Technology) for 1 h at room temperature. Immunoreactive bands were visualized by Imaging System (Tanon Science & Technology) after adding chemiluminescent HRP substrate (Millipore Corporation). Primary antibodies used in this study were listed in Supplementary Table 1.

### Co-immunoprecipitation (Co-IP)

Cellular proteins were extracted with the Cell lysis buffer for IP (Beyotime). Proteins (500 μg) were incubated overnight at 4 °C with a rabbit anti-p27 antibody, or a control IgG. The immune complexes were immuno precipitated with Protein A/G Plus-Agarose beads (Santa Cruz), washed for three times with TBST, subjected to 12% SDS-PAGE, and detected with anti-CDK2, anti-Cylin A, or anti-p27 antibodies, respectively.

### Flow cytometry

The percentage of apoptosis was determined using annexin-V fluorescein isothiocyanate (FITC) and propidium iodide (PI) apoptosis detection kit (BD Biosciences) according to the manufacturer’s instructions. Percentage of apoptotic cells (annexin+/PI+) was analyzed by flow cytometry (BD Biosciences).

The distribution of cell cycle was determined by staining permeabilized cells with PI dye after treatment with EP (Sigma) or Dasatinib (Selleckchem) for 24 h. Cells were fixed and permeabilized with 70% alcohol at 4 ℃ and then washed twice with PBS. Cells were incubated with RNase A for 30 min and then stained with PI at room temperature in the dark. For Ki67 expression, cells were permeabilized and fixed, then washed with PBS. Cells were incubated with anti-Ki67-FITC or FITC isotype (BD Biosciences) for 1 h at 4 ℃. Before flow cytometry analysis, 10 μg/ml of PI was added to the cell suspension. Nuclear DNA content was assessed with PI and analyzed by flow cytometry (BD Biosciences). Ki67 negative cells were defined as G0 cells^9^.

### Immunostaining and fluorescent microscopy

Cells on slides were dried, fixed and permeabilized with Cytofix/Cytoperm reagents (BD Biosciences) and blocked with a buffer consisting of 0.1% saponin and 5% serum (the type of serum corresponding to the secondary antibody). Cells were costained with primary antibody (Supplementary Table 1) for 1 h at room temperature. After washing with TBST, cells were incubated with conjugated secondary antibodies at 1:100 dilution. Slides were washed for three times with TBST, stained with 50 ng/ml DAPI (4′, 6-diamidino-2-phenylindole, Solarbio), air-dried at 4 °C in the dark, and mounted in ProLong^®^ Gold anti-fade reagent (Life Technologies) before being viewed under fluorescent microscope (Zeiss, Germany). The levels of colocalization were quantitatively expressed by the Pearson’s correlation coefficient (Rr) using WCIF ImageJ software^50,63^.

### In vivo experiments

Ethical approval for this in vivo study was obtained from the ethics committee of Tianjin Medical University Cancer Institute and Hospital. For EP toxicity assay, young BALB/C mice (four to six weeks old) with similar weight were randomly and equally divided into four groups. Mice were intraperitoneal injected NaCl, 40 mg/kg EP, 80 mg/kg EP, or 160 mg/kg EP, respectively, twice/per day, for 39 days and weighed every 3 days. For tumorigenicity assay, 6 × 10^6^ A20 cells were injected into young BALB/C mice (four to six weeks old). When tumor was palpable, mice were randomly and equally divided into three groups and undergone intraperitoneal injection of NaCl, 40 mg/kg EP, or 80 mg/kg EP twice/per day for 30 days. Over a 30-day period, tumor volume was measured and calculated by the formula *V* = 0.5 × *L* × *W*^2^ (*V*, volume; *L*, length, and *W*, width) for comparison of tumor growth. Mice were killed if profoundly ill and were scored as a death in surviving analysis. At day 38, surviving mice were euthanized by cervical dislocation.

### Statistical analysis

Statistical analysis was performed using GraphPad Prism 5.0 software. Data were shown as either mean ± SEM or mean ± SD; comparisons between groups were analyzed with either the unpaired student *t*-test or one-way ANOVA. The effect of EP on tumor-bearing mice survival was analyzed using log rank test and presented as Kaplan–Meier curves. The significant threshold was set up at 0.05. *P < 0.05, **P < 0.01, and ***P < 0.001.

## Supplementary information

Supplementary Figures

Supplementary Table
