# Supplementary material for: Blockade of HMGB1 signaling pathway by ethyl pyruvate inhibits tumor growth in diffuse large B-cell lymphoma
Source: Cell Death Dis. 2019 Apr 15;10(5):330. doi: 10.1038/s41419-019-1563-8 (PMC6465275; doi:10.1038/s41419-019-1563-8)
Supplement: Supplementary file 1 — Supplementary Figures [file 41419_2019_1563_MOESM1_ESM.docx]

TMD8

Su-4

Su-10

**p-Akt^S-473^**

p-ERK1/2

ERK


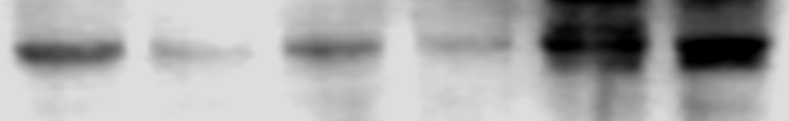

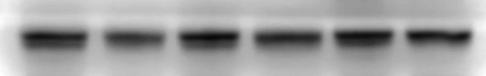

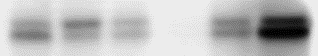

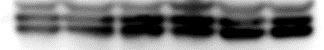


OCI-Ly7

DoHH2

OCI-Ly3

GCB

ABC


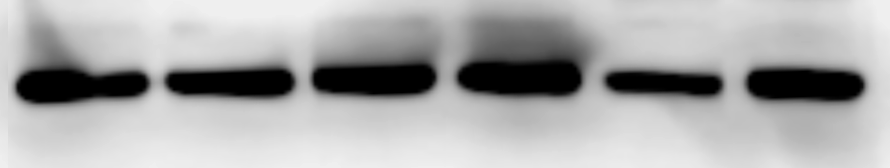


α-tubulin

AKT

STAT3

p-STAT3^Y705^


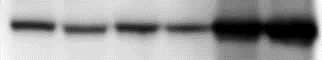

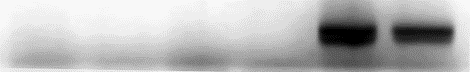


A

B

C

D

E

**Suppl Figure 1. HMGB1 stimulates proliferation of GBC-type DLBCL cells**. (A) Expression of survival signaling proteins in GCB and ABT-DLBCL cells were detected by Western blotting. (B-E) Effects of HMGB1 and/or EP on DLBCL cell proliferation was determined by a cell counter.

Suppl Figure 2. EP-induced inhibition on A20 cell growth. A20 cells

were treated with EP for 48 hours and EP-mediated growth

inhibition was determined by MTT test. IC50 was calculated by

Log (inhibitor) vs. response. Data shown are mean ± SD, n=8.


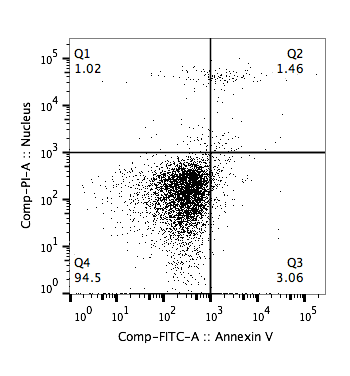

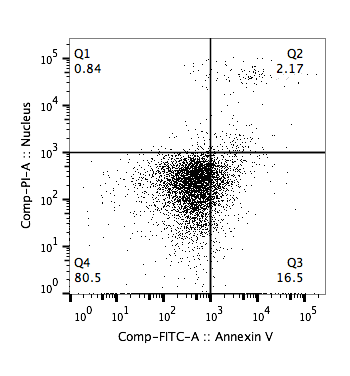

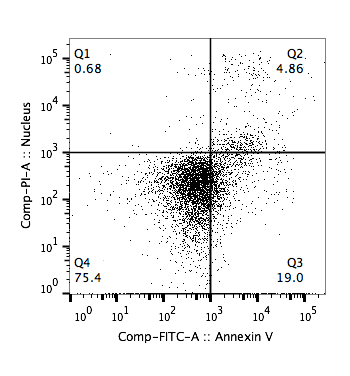

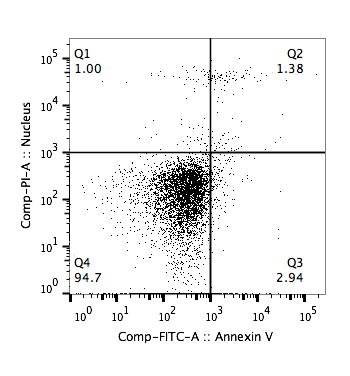


Annexin V

PI

EP (mM)

2

4

8

0

A

B

D

E

F

C


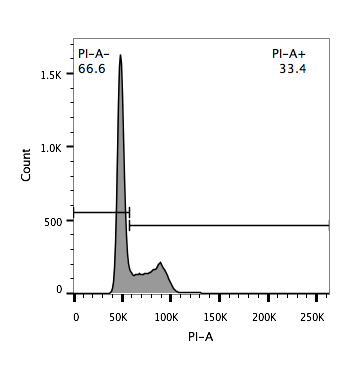

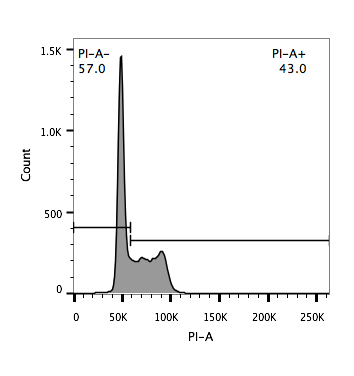


PI

cell number

EP (mM)

2

4

8

0

**G0/G1=57.0%**

**G0/G1=66.6%**


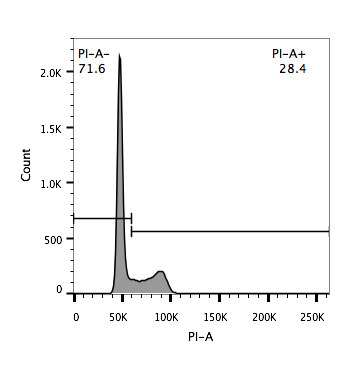

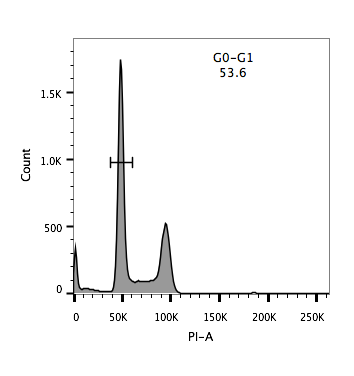


**G0/G1=71.6%**

**G0/G1=53.6%**

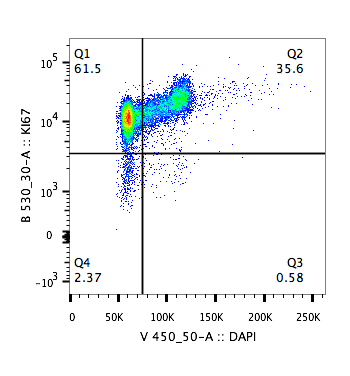

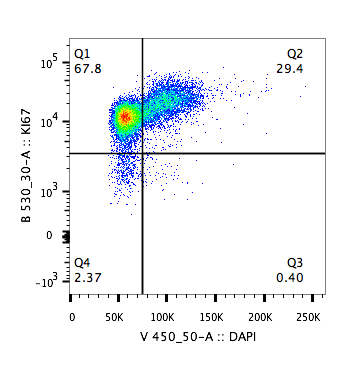


PI

Ki67

EP (mM)

2

0

**Suppl. Figure 3. Flow cytometry analysis of EP on apoptosis and cell cycle progression.** GCB-DLBCL Su-8 cells were cultured with or without indicated concentrations of EP for 24 hrs.

(A and C) Apoptotic cell death was measured by flow cytometry after staining with Annexin V-FITC and PI. (B and D) Cell-cycle distribution analysis by quantitation of DNA content. Cells were stained with PI and DNA content was analyzed by flow cytometry. (E and F) Cell-cycle analysis with Ki67 and PI. Permeabilized cells were stained with Ki67-FITC and PI and DNA content was analyzed by flow cytometry. The percentages of cells within each cell cycle phase are depicted by density plots. (C, D and F) Data shown (mean ± SD) are from three independent experiments. The significant difference between EP treated and control were analyzed by the Student *t*-test.

Cytoplasmic

Colocalization


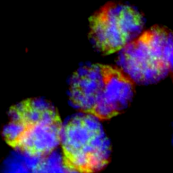

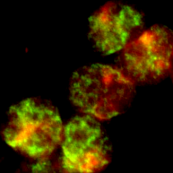

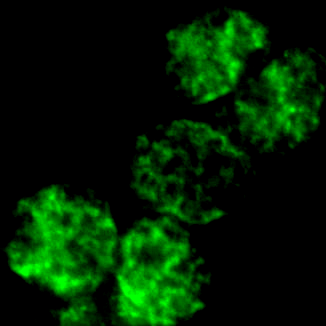

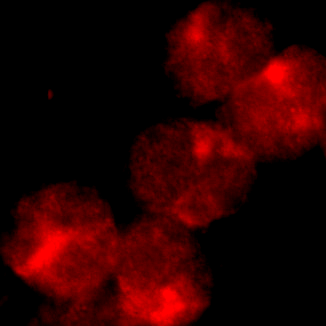


**p27**

**CDK2**

**Ctrl**

**Merge**

**DAPI**

**EP**


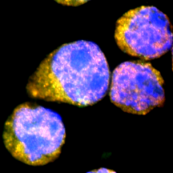

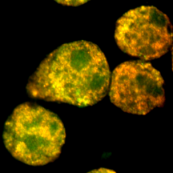

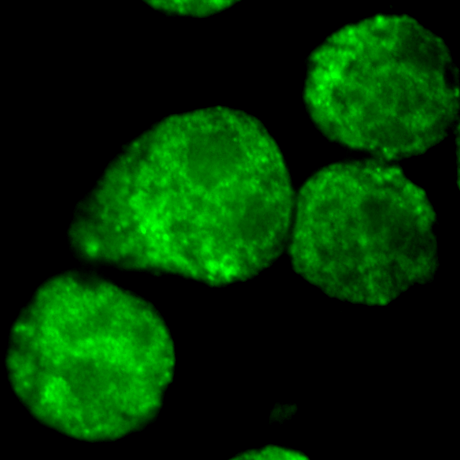

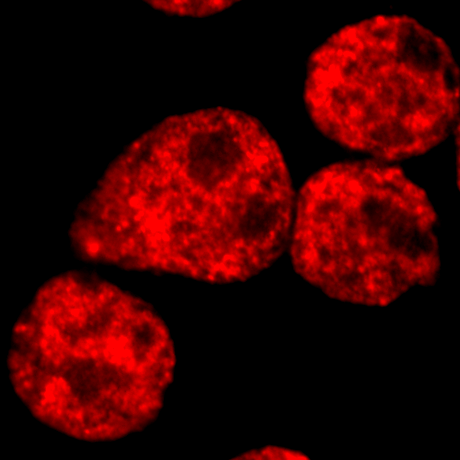


**Su-8**

A


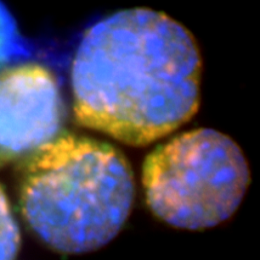

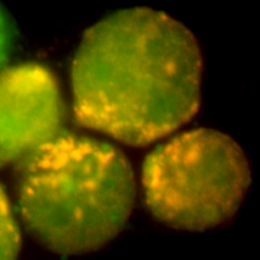

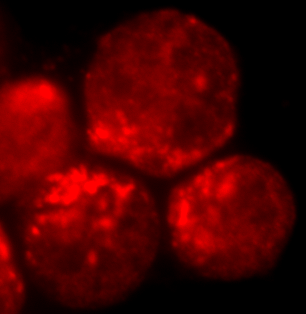

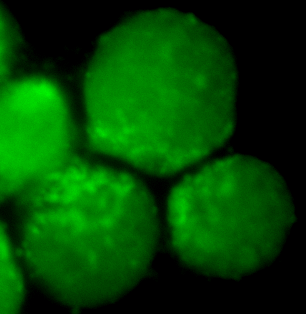


**Ctrl**

**EP**

**Ly-3**


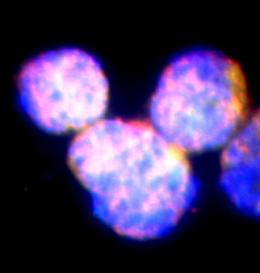

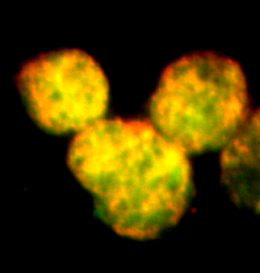

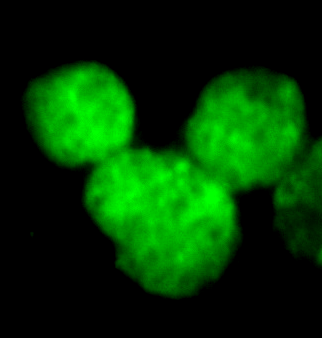

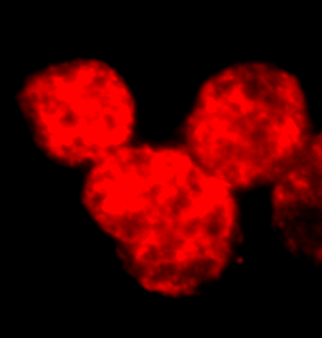


B

**Suppl. Figure 4. Immunofluorescent determination of colocalization of p27 and CDK2 in Su-8 and Ly-3 cells.** After treatment, cells on slides were co-stained with mouse anti-CDK2 (red)/rabbit anti-p27 (green) antibodies. Secondary antibodies for co-staining were FITC-conjugated goat anti-rabbit IgG and Rhodamine-conjugated goat anti-mouse IgG. White arrows indicate cytoplasm and red arrows indicate colocalization of p27 and CDK2.


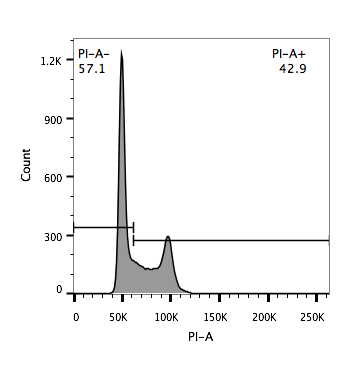

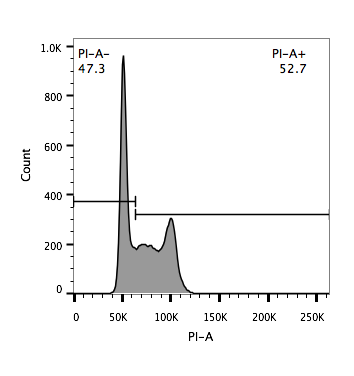

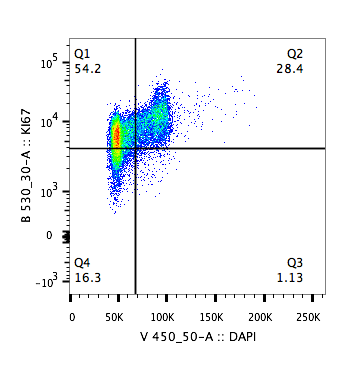

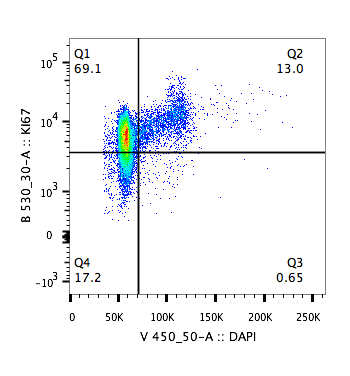


**Suppl. Figure 5. The role of SRC phosphorylation in HMGB1-mediated cell**

**cycle progression.** (A) Effects of EP on phosphorylated Src and ERK and levels

of CDK2 and p27 in GCB-DLBCL Ly-7 and Su-8 cells. Cells were treated with

2mM EP for 6 or 24 hrs. (B) Effect of Src inhibitor Dasatinib on phosphorylated

Src and ERK, and levels of CDK2 and P27 in GCB-DLBCL cells. Cells were treated

with 1 µM Dasatinib for 2 or 4 hrs. (C and D) Dasatinib-induced G0/G1 arrest.

Su-8 cells were treated with Dasatinib for 24 hrs. Cell cycle was analyzed by

DNA content analysis. (E and F) Effect of Dasatinib on G1 arrest. Cells were

stained with Ki67 and PI. G0 phase was defined as the Ki67 negative population.


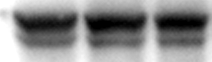

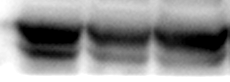

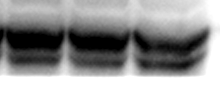

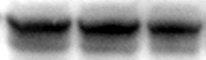

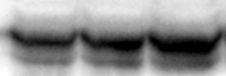

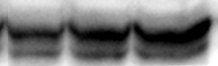

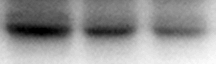

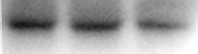

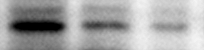

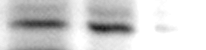

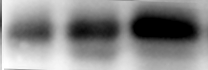

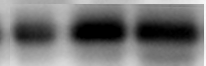

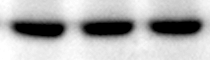

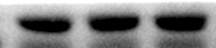


**p-Src**

**Src**

**p-ERK**

**ERK**

**CDK2**

**p27**

**β-actin**

**EP 2mM**

**Time (h)**

**6**

**24**

**0**

**6**

**24**

**0**

**Su-8**

**Ly-7**

**Dasatinib 1μM**


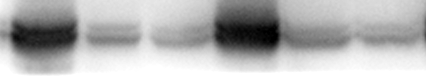

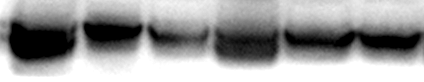

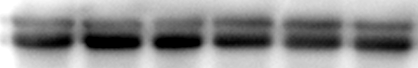

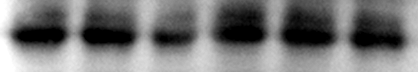


**0**

**2**

**4**

**0**

**2**

**4**

**Ly-7**

**p-Src**

**Src**

**p-ERK**

**ERK**

**Time (h)**

**Su-8**

**CDK2**

**p27**


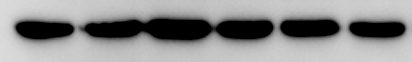


**β-actin**


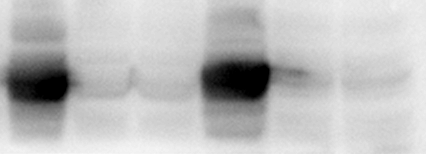

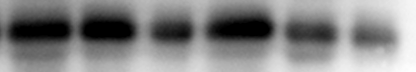


PI

Cell number

Dasatinib (μM)

1

0

PI

Ki67

Dasatinib (μM)

1

0

A

B

D

C

F

E

**G0/G1=47.3%**

**G0/G1=57.1%**
