# Supplementary material for: Blockade of HMGB1 signaling pathway by ethyl pyruvate inhibits tumor growth in diffuse large B-cell lymphoma
Source: Cell Death Dis. 2019 Apr 15;10(5):330. doi: 10.1038/s41419-019-1563-8 (PMC6465275; doi:10.1038/s41419-019-1563-8)
Supplement: Supplementary file 2 — Supplementary Table [file 41419_2019_1563_MOESM2_ESM.docx]

Supplementary Table 1 List of primary antibodies and chemicals.

| Name of antibody | Type | Company | Cat No | Application | Dilution |
| --- | --- | --- | --- | --- | --- |
| HMGB1 | Rabbit | Abcam | ab18256 | WB | 1:3000 |
| HMGB1 | Chicken | IBL | ST326052233 | Neutralizing | 10 µg/ml |
| transferrin | Mouse | Santa Cruz | sc-52256 | WB | 1:500 |
| p-Src | Rabbit | Cell Signaling |  | WB | 1:3000 |
| Src | Rabbit | Cell Signaling | 2109 | WB | 1:3000 |
| P-SYK | Rabbit | Cell Signalling | 2710 | IF | 1:100 |
| p-ERK1/2 | Rabbit | Cell Signaling | 4377 | WB | 1:3000 |
| ERK1/2 | Rabbit | Cell Signaling | 9102 | WB | 1:3000 |
| p-ERK1/2 | Rabbit | Cell Signalling | 9101 | IF | 1:100 |
| TLR9 | Mouse | Abcam | Ac12121 | IF | 1:100 |
| p27 | Rabbit | Proteintech | 25614-1-AP | WB | 1:3000 |
|  |  |  |  | IP | 1:100 |
|  |  |  |  | IF | 1:100 |
| CDK2 | Mouse | Santa Cruz | sc-6248 | WB | 1:500 |
|  |  |  |  | IF | 1:20 |
| Cyclin A | Mouse | Santa Cruz | sc-271682 | WB | 1:500 |
| β-actin | Mouse | Sigma | A5316 | WB | 1:10000 |
| HMGB1 protein | Human | R&D | 1690-HMB-050 | Treatment | 200ng/ml |
| Ethyl pyruvate (EP) |  | Sigma | E47808 | Treatment | 2mM; 40 or 80 mg/Kg |
| Dasatinib |  | Selleck | S1021 | Treatment | 1μM |
